# Bioclipse-R: integrating management and visualization of life science data with statistical analysis

**DOI:** 10.1093/bioinformatics/bts681

**Published:** 2012-11-23

**Authors:** Ola Spjuth, Valentin Georgiev, Lars Carlsson, Jonathan Alvarsson, Arvid Berg, Egon Willighagen, Jarl E.S. Wikberg, Martin Eklund

**Affiliations:** ^1^Department of Pharmaceutical Biosciences, Uppsala University, SE-751 24 Uppsala, Sweden, ^2^AstraZeneca Research and Development, SE-431 83 Mölndal, Sweden and ^3^Department of Bioinformatics - BiGCaT, Maastricht University, NL-6200 MD Maastricht, The Netherlands

## Abstract

**Summary:** Bioclipse, a graphical workbench for the life sciences, provides functionality for managing and visualizing life science data. We introduce Bioclipse-R, which integrates Bioclipse and the statistical programming language R. The synergy between Bioclipse and R is demonstrated by the construction of a decision support system for anticancer drug screening and mutagenicity prediction, which shows how Bioclipse-R can be used to perform complex tasks from within a single software system.

**Availability and implementation:** Bioclipse-R is implemented as a set of Java plug-ins for Bioclipse based on the R-package rj. Source code and binary packages are available from https://github.com/bioclipse and http://www.bioclipse.net/bioclipse-r, respectively.

**Contact:** martin.eklund@farmbio.uu.se

**Supplementary information:** Supplementary data are available at *Bioinformatics* online.

## 1 INTRODUCTION

Bioclipse (Spjuth *et al.*, 2007, 2009) and R (R Development Core Team, 2011) are free and open source software systems for the life sciences and for statistics, respectively. Although Bioclipse has excellent functionality for managing, editing and visualizing chemical and biological information, it lacks data analytical capabilities. R, on the other hand, is outstanding for statistics and data analysis, but does not provide functionality for interactively reading, editing and visualizing chemical structures or protein sequences. In this applications note, we describe and demonstrate Bioclipse-R, a set of Bioclipse plug-ins that link Bioclipse to R and provide access to R methods from within Bioclipse.

The Bioconductor project (Gentleman *et al.*, 2004) has equipped R with tailor made functionality for biology. However, Bioconductor is primarily targeted at genomics and has no functionality for chemistry. The R package rcdk adds core cheminformatics functionality to R but is not focused on providing a rich graphical user experience with functionality for editing and visualizing chemical data in 2D and 3D. Other tools for integrating R in graphical user interfaces with applications in biology and chemistry include the workflow tools Konstanz Information Miner (KNIME) (Warr, 2012) and Taverna (Oinn *et al.*, 2004). These provide nodes in a workflow that are capable of containing R code, but they are focused on constructing reproducible workflows and do not expose R in a workbench environment where it can be used programmatically and in combination with other tools in a more investigative fashion. In contrast to these tools, Bioclipse-R integrates R with the rich client Bioclipse and, thus, provides cross-fertilization between R and all other functionality in Bioclipse, for example, advanced visualizations, help systems and semantic data management (Willighagen *et al.*, 2011).

We demonstrate the synergy between Bioclipse and R by constructing a decision support system for virtual screening and mutagenicity testing of new cancer drugs. Constructing this system without the integration between Bioclipse and R would require extensive efforts of copying data between various software tools and file formats, but is almost trivial with Bioclipse-R. Here, we outline the basic steps of the demonstration and refer to http://bioclipse.net/bioclipse-r for a detailed step-by-step description. The operations can be performed both in the Bioclipse Scripting Language (BSL) and in Bioclipse’ graphical user interface.

## 2 DEMONSTRATION

This section describes the workflow for constructing the decision support system for virtual screening and mutagenicity testing of new cancer drugs. It consists of three parts: (i) construction of a quantitative structure-activity relationships (QSAR) model for anticancer activity; (ii) construction of a QSAR model for mutagenicity; and (iii) using the models constructed in (i) and (ii) to perform a virtual screen of the DrugBank approved drugs database for non-mutagenic compounds with anticancer activity.

### 2.1 Cancer cell line growth inhibition modelling

**Fig. 1.**
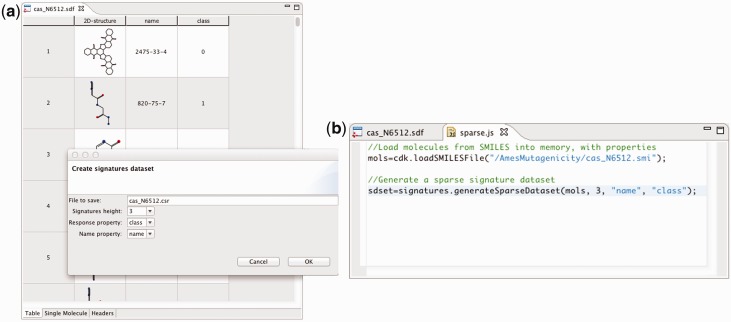
(**a**) Dataset creation by calculating descriptors using the graphical components in Bioclipse. (**b**) The same dataset calculation using a script in the Bioclipse Scripting Language. This is a part of the demonstration example *Mutagenicity modelling*; the full tutorial is available in the Supplementary Material

**Fig. 2.**
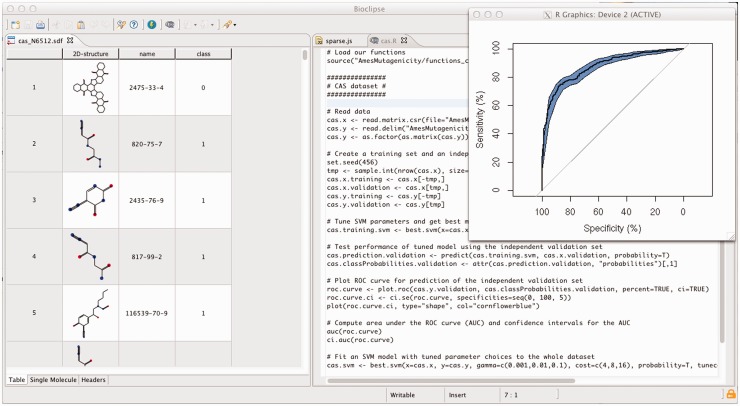
Screenshot of Bioclipse with the R-script for building the predictive model in the *Mutagenicity modelling* with the ROC curve shown in the plot. The full tutorial is available in the Supplementary Material at http://bioclipse.net/bioclipse-r

The NCI60 is a panel of 59 cancer cell lines developed by the National Cancer Institute (NCI), which is used for screening of new anticancer drug candidates (see, e.g. Shoemaker, 2006). Using a set of 3515 chemical compounds screened against the glioblastoma cell line U251, we built a QSAR model to predict whether a new compound has anticancer activity according to the following workflow:

1. *Import and inspect molecules*. The chemical structures were downloaded as a Simplified Molecular-Input Line-Entry System (SMILES)-formatted file and imported into a new project in Bioclipse. Bioclipse uses the Chemistry Development Kit (CDK) (Steinbeck *et al.*, 2003) as the cheminformatics library for input, output and internal representation of chemical structures, as well as for structure diagram generation and rendering. The Bioclipse Molecules Table (Figs 1a and 2) and the chemical structure editor JChemPaint (Krause *et al.*, 2000) were used to inspect the chemical structures.
2. *Generate 3D coordinates*. We used the Balloon (Vainio and Johnson, 2007) Bioclipse plug-in to generate 3D coordinates for the molecules so we could view the molecules in the integrated 3D viewer Jmol (Hanson, 2010) and compute descriptors that require 3D coordinates.
3. *Compute descriptors for the molecules*. Constitutional, electronic, atomic, molecular and topological descriptors were computed with the Bioclipse-QSAR plug-in (Spjuth *et al.*, 2010), which can be used both form a graphical user interface and through a scripting interface (Fig. 1). The descriptors were calculated for compounds in their neutral state.
4. *Build and validate QSAR model*. Using R from within Bioclipse, we split the U251 dataset into a training set containing 2515 molecules and a test set containing 1000 molecules (the split was done by random selection). Using the training data, we built a random forest model to discriminate between compounds with and without anticancer activity [the R-package ‘randomForest’ (Liaw and Wiener, 2002) with the default parameter settings was used]. We then used the model to predict the activity of the compounds in the test set, resulting in an area under the receiver operating characteristics curve (AUC) of 77.8% (95% confidence interval: 74.9–80.7%), indicating relatively good predictive performance [the R-package ‘pROC’ (Robin *et al.*, 2011) was used for computing the AUC]. For details and R-code, see http://pele.farmbio.uu.se/bcr/bcr-screening.html.

### 2.2 Mutagenicity modelling

As it is not uncommon for anticancer drugs to be mutagenic, we wanted to be able to test compounds predicted to have anticancer activity for mutagenicity. We, therefore, constructed a QSAR model predicting mutagenicity based on a set of 6504 compounds tested with the Ames assay (Hansen *et al.*, 2009). This was done as follows:

1. *Import and inspect molecules*. (Analogous to 1).
2. *Compute signature descriptors and set-up dataset*. Signature descriptors (Faulon *et al.*, 2003) are of a different nature than molecular descriptors calculated with Bioclipse-QSAR in 3. The signature descriptors are provided by the Bioclipse-Decision Support (DS) plug-in (Spjuth *et al.*, 2011), instead, and because they do not require 3D coordinates, the analogue of 2 is skipped.
3. *Build and validate QSAR model*. We used R within Bioclipse to split the Ames dataset into a training set and a test set, containing 5504 and 1000 molecules, respectively (by random selection). We then used the R-package ‘e1071’ (Dimitriadou *et al.*, 2010) to fit a support vector machine with a Gaussian kernel to the training set to discriminate mutagenic from non-mutagenic compounds. The parameters 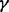 and 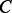 in the Gaussian kernel function were optimized using a grid search, where 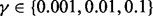 and 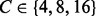. Three-fold cross-validated classification error was used as objective function in the grid search. Using the support vector machine model optimized on the training data to predict the mutagenicity in the test set, we achieved an AUC of 87.85% (95% confidence interval 85.72–89.98%), indicating good predictive performance. For details and R-code, see http://pele.farmbio.uu.se/bcr/bcr-mutagenicity.html.

### 2.3 Virtual screening and mutagenicy prediction

**Fig. 3.**
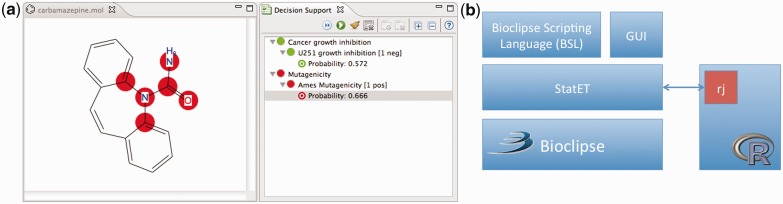
(**a**) Screenshot of Bioclipse showing the chemical structure of carbamazepine, which was predicted to inhibit cancer cell growth and to be mutagenic. The circles highlight the parts of the chemical structure predicted to cause the mutagenicity (see Spjuth *et al.*, 2011). (**b**) Architecture of the Bioclipse-R integration. Bioclipse makes use of the StatET plug-ins for interacting with R using the R-package rj (Wahlbrink and Verbeke, 2011), and it makes R available both via the user interface as well as the BSL

Using the two QSAR models for growth inhibition and mutagenicity prediction constructed in Sections 2.1 and 2.2, we screened the subset of DrugBank (Knox *et al.*, 2011) with approved drugs:

1. *Screen approved compounds from DrugBank*. Using the BSL and the Bioclipse-DS, JChemPaint and Jmol Bioclipse plug-ins, we iterated over the molecules in the DrugBank approved dataset, generated 3D coordinates for the compounds, computed descriptors, and predicted each molecule’s anticancer activity (using the QSAR model constructed in Section 2.1) and mutagenicity (using the QSAR model constructed in Section 2.2). Using a BSL script, we generated a table sorted on growth inhibition (Fig. 2).
2. *Display screening hit in 2D and 3D*. We inspected the top screening hits in 2D using the Bioclipse Molecules Table, which also permits easy access to DrugBank information for these compounds using hyperlinks embedded in the table that open in the internal browser in Bioclipse. Using the Balloon conformer generation, we could generate 3D coordinates and inspect the screening hits in 3D using the Jmol component in Bioclipse. The focus of this article is the integration of Bioclipse and R, and not the results of the screening *per se*. However, we may note that three of the top five hits from the screening model are drugs used in cancer treatment (plicamycin, oxaliplatin and daunorubicin, see Figure 2 and http://pele.farmbio.uu.se/bcr/bcr-vscreen.html), tentatively indicating that the growth inhibition screening model does a decent job in ranking molecules.
3. *Interpret predictions*. To interpret the results, we re-ran the predictions in Bioclipse Decision Support. When using the growth inhibition and mutagenicity models in Bioclipse Decision Support, the substructure that contributed the most to the prediction of a given compound’s bioactivity is highlighted. The highlighted substructure is, thus, the site where structural modification would affect the predicted bioactivity the most (Fig. 3a). This interpretation of non-linear models is based on the method by Carlsson *et al.* (2009) and allows for guidance when exploring different hypotheses of how structural changes of molecules would affect their predictions (a screencast demonstrating this functionality in further detail is available at http://pele.farmbio.uu.se/bcr/bcr-vscreen.html).

## 3 IMPLEMENTATION AND AVAILABILITY

A set of plug-ins for Bioclipse were implemented in Java, adding a bridge to R. The plug-ins build on the StatET project (http://www.walware.de/goto/statet), which in turn uses the R-package rj (Wahlbrink and Verbeke, 2011) (see Fig. 3b). Bioclipse maintains a persistent connection with R, and allows for executing commands and scripts and retrieving result back into Bioclipse, with functionality exposed both in the graphical user interface as well as from BSL.

Bioclipse-R is available for the operating systems Windows, Linux and Mac OS X. Source code and binary packages are available under the Eclipse Public License (http://www.eclipse.org/org/documents/epl-v10.php) from https://github.com/bioclipse and http://www.bioclipse.net/bioclipse-r, respectively.

## 4 CONCLUSION

We show how we can conduct advanced tasks from within a single software platform by leveraging the functionality in Bioclipse and R. The demonstration shows the cross-fertilization between R and numerous Bioclipse components (CDK, Balloon, Bioclipse-QSAR, Bioclipse-DS, JChemPaint and Jmol). It also shows how Bioclipse-R allows for easy access to statistical models fitted in R from a graphical user interface as well as from BSL scripts, thus allowing for automation and exchange of complete Bioclipse-R workflows. Bioclipse-R also enriches the bioinformatics functionality in Bioclipse through access to Bioconductor via R. The plug-in-based architecture of Bioclipse makes extensions simple, and future development of Bioclipse-R will include a user-friendly graphical interface for performing statistical analyses.

*Funding:* Support from Uppsala University (KoF 07), Swedish VR-2011-6129, and the Swedish strategic research program eSSENCE.

*Conflict of Interest:* none declared.
